# The *Dioscorea* Genus (Yam)—An Appraisal of Nutritional and Therapeutic Potentials

**DOI:** 10.3390/foods9091304

**Published:** 2020-09-16

**Authors:** Jude E. Obidiegwu, Jessica B. Lyons, Cynthia A. Chilaka

**Affiliations:** 1 National Root Crops Research Institute, Umudike, Km 8 Umuahia-Ikot Ekpene Road, P.M.B 7006 Umuahia, Abia State, Nigeria; 2 Department of Molecular and Cell Biology and Innovative Genomics Institute, University of California, Berkeley, 142 Weill Hall #3200, Berkeley, CA 94720-3200, USA; jblyons@berkeley.edu; 3 Institute of Pharmacology and Toxicology, Julius Maximilian University of Würzburg, Versbacher Straβe 9, 97078 Würzburg, Germany; adaku80@yahoo.com or

**Keywords:** yam, *Dioscorea*, nutritional composition, bioactive compounds, therapeutic potential

## Abstract

The quest for a food secure and safe world has led to continuous effort toward improvements of global food and health systems. While the developed countries seem to have these systems stabilized, some parts of the world still face enormous challenges. Yam (*Dioscorea* species) is an orphan crop, widely distributed globally; and has contributed enormously to food security especially in sub-Saharan Africa because of its role in providing nutritional benefits and income. Additionally, yam has non-nutritional components called bioactive compounds, which offer numerous health benefits ranging from prevention to treatment of degenerative diseases. Pharmaceutical application of diosgenin and dioscorin, among other compounds isolated from yam, has shown more prospects recently. Despite the benefits embedded in yam, reports on the nutritional and therapeutic potentials of yam have been fragmented and the diversity within the genus has led to much confusion. An overview of the nutritional and health importance of yam will harness the crop to meet its potential towards combating hunger and malnutrition, while improving global health. This review makes a conscious attempt to provide an overview regarding the nutritional, bioactive compositions and therapeutic potentials of yam diversity. Insights on how to increase its utilization for a greater impact are elucidated.

## 1. Introduction

**Figure 1:**
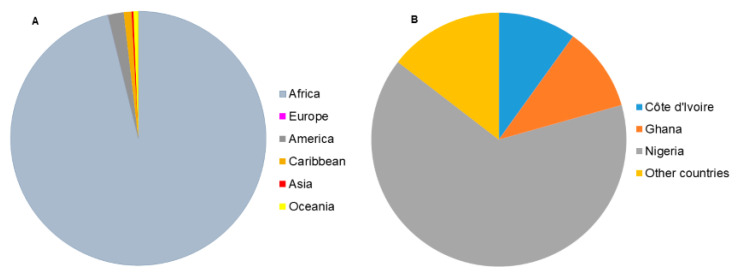
(**A**) Global distribution of yam production in 2018 (Africa 96.2%, America 2.0%, Caribbean 1.0%, Oceania 0.6%, Asia 0.2%, Europe 0%), (**B**) Top yam producing countries in 2018 (Nigeria—65.9%, Ghana–10.7%, Côte d’Ivoire—9.9%, other countries—14.5%) [11].

The nomenclature “Yam” applies to members of the *Dioscorea* genus of the *Dioscoreaceae* family within the order *Dioscoreales* [1]. The yam crop was initially referred to as Inhame by New Guinea users who predominantly used them as a starchy food source [2]. In the course of the 16th century, French sailors erroneously changed the name from Inhame to Igname. Within this period, English seamen called the crop “yam.” Yam was a source of food for enslaved people during their East to West historic migration [2]. The roots, tubers and rhizomes of yams have been used since pre-historic times by aboriginal peoples as a food, as well as for traditional medicine [3]. *Dioscorea* comprises over 600 species, with varying global distribution across Africa, Asia, Latin America, the Caribbean and Oceania (Figure 1A) [4]. Among the wide species reported, only about 10 species are estimated to have been domesticated across Africa, Asia and Latin America for food and income generation [5]. Yam plants have unique climbing and twining vines that sprout from their characteristic rhizomes or tubers. These rhizomes and tubers most often serve as photosynthetic sinks for starch and other secondary metabolites [6]. 

Across different ethnic communities and geographic regions, diverse species of *Dioscorea* have been adopted within different habitation as a food source due to the high nutritional benefits and therapeutic values toward treatment and cure of certain health problems [7,8]. Whilst yam is one of the most important staple root and tuber crops worldwide, it is still classified as an orphan crop because it is highly underutilized and receives little investment or/and research attention toward crop improvement. Yam plays a significant role in food security, medicine and economy in the developing countries. Its importance places it as the fourth most essential and utilized root and tuber crop globally after potatoes (*Solanum* spp.), cassava (*Manihot esculenta*) and sweet potatoes (*Ipomoea* spp.) and the second in West Africa after cassava [9,10]. This is evident in annual global production, especially in West Africa (Figure 1B). In 2018, the Food and Agricultural Organization (FAO) of the United Nations reported a worldwide production of approximately 72.6 million tons over 8.7 million hectares of harvested area at a yield rate of 83515 hg/ha, with Africa contributing 97.1% of global production [11].Remarkably, among the African nations, three countries (Nigeria—67.4%, Ghana—11.1%, Côte d’Ivoire—10.3%) in the west recorded the highest proportion of production, although the production increase (85.1% between 2000–2018) in Africa is attributed mainly to the increase in the area of yam field into marginal lands and non-traditional yam growing areas [12]. While *D. alata* originated in Asia and is the most globally cultivated yam species, *D. rotundata* represents a great significance in respect to production volume in the West of Africa, followed by *D. alata* and *D. cayenensis* [13]. Statistics have shown evidence of an annual production increase of yam between 2011 and 2018 in several countries on the African continent including Cameroon, Central African Republic, Côte d’Ivoire, Gabon, Ghana, South Sudan and United Republic of Tanzania [11]. This could be ascribed to its serving as a major food source and cash crop, thus, combating malnutrition, food insecurity and poverty. In addition, the significance of yam in the cultural, social and religious environment of West Africa cannot be overemphasized [4,14,15]. Its symbolism as king of crops is manifested in its use in ceremonies such as those for fertility and marriages, as well as an annual festival held to celebrate its harvest. Importantly, the cultural and linguistic diversity that cuts across West Africa has no influence on the beliefs, social values and religious practices attached to the yam crop.

Yam’s potential as a source of food is attributed to its high levels of carbohydrates including fiber, starch and sugar, contributing about 200 dietary calories per person per day to 300 million people in the tropics [12]. It also provides other nutritional benefits such as proteins, lipids, vitamins and minerals [16]. According to International Institute of Tropical Agriculture (IITA), the global annual consumption of yam is placed at 18 million tons, with 15 million tons only in West Africa amounting to about 61 kg per capita in the region [17]. In West Africa, yam tuber may be eaten boiled, fried, baked or roasted in combination with tomato stew, sauces and in some cases, typically in poor rural communities, with traditional palm oil. The tuber may also be pounded into moldable dough which is consumed with traditional African soups. The consumption of raw yam tubers of species *D. soso*, *D. nako* and *D. fandra* in Madagascar has also been reported [12]. In Asia, especially Japan and China, *D. japonica* and *D. polystachya*, usually eaten raw, can also be grated and used as an ingredient in tororo udon/soba noodles [18,19].

In the quest to identify other benefits of *Dioscorea* species, studies have revealed yam therapeutic potentials as a result of its bioactive compound content. A bioactive compound is defined as a substance that can exert biological effect, thus, causing a reaction or triggering a response in a living tissue [20]. A study on seven different varieties of yams (*Dioscorea* spp.) reported reasonable quantities of these compounds including flavonoids, phenols, saponins, tannins and alkaloids [21]. Another study described the pharmacological activities of yam peptides and proteins such as antioxidant, immunomodulatory, estrogenic, angiotensin I-converting enzyme inhibiting, carbonic anhydrase and trypsin inhibiting, chitinase, anti-insect, anti-dust mite, lectin and anti-proliferative activities [22]. These authors reported the therapeutic potentials of peptides and proteins isolated from several species of yams including *D. alata*, *D. cayenensis*, *D. japonica*, *D. pseudojaponica* and *D. polystachya* (formerly known as *D. opposita* or *D. batatas*), as well as the possible clinical applications for the treatment of inflammatory diseases, cardiovascular diseases, aging disorders, menopause, cancers and osteoporosis. Furthermore, the use of different species of *Dioscorea* for birth control and skin infections has been reported [23,24]. Nashriyah et al. [25] reported the use of *D. hispida* in cosmetics for pigmentation remedy. This is not surprising as since time immemorial, the utilization of natural products with therapeutic potentials including mineral, plant and animal substances as main sources of drugs have existed [26]. This long standing historic use of plants as therapeutic resources serves as a proof of their efficacy. The diversity in yams has the potential to enrich the human body with starch and energy [27], as well as supplemental metabolites while serving as a source for medicinal use at level of traditional therapeutics and industrial medical pharmacy. The therapeutic potential of yam is of interest especially in developing countries where a majority of the population lacks access to standard health care, which even when available is far beyond the reach of many locals due to the financial burden, thus, yam may contribute to providing health benefits beyond its nutritive values.

While there are pharmacological prospects of yam, its antinutritional components cannot be overlooked. The utilization of some species of yam, such as *D. bulbifera* and *D. hispida,* have been hindered due to the bitter taste caused by the presence of furanoid norditerpenes (diosbulbin) and dioscorine, respectively [28,29]. However, in the context of extreme food scarcity, processing such as soaking, boiling and roasting are used to reduce or eliminate the bitterness. In addition, diosbulbin and dioscorine have been reported to trigger fatal paralysis of the nervous system [30]. This is evident in the utilization of these yam extracts in the preparation of arrow poison or sedative drugs often used for hunting in different countries including Malaysia, Indonesia, South Africa and Bangladesh [4,25]. In addition, other toxic compounds and allergens such as oxalate, saponin, phytic acid, tannin and histamine have been reported [31], with some species such as *D. hispida* having cyanide [32]. Shim and Oh [33] described histamine as one of the major compounds that induce allergic reactions such as an itch on the skin. Although histamine may be the principal allergen in yam, studies have also reported the potential of dioscorin from *D. batatas* (presently known as *D. polystachya*) to cause allergic reaction [34].

To utilize the full potential of *Dioscorea* spp. given their contributory role in food security as a staple crop to a large number of the world’s population and their beneficial health effects, the need for harmonization of indigenous and scientific knowledge of this crop becomes imperative. Such knowledge will help to promote its utilization, thus contributing to the attainment of United Nations (UN) sustainable development goals (SDGs 1, 2 and 3) especially in the developing countries. In this framework, the present review aims to reveal the global importance of yam by providing a comprehensive report on the nutritional and bioactive composition of *Dioscorea* species. In addition, the review will highlight the therapeutic benefits and impact on human health associated with the consumption of yam, as well as future perspectives of yam production, utilization and research.

## 2. Yam Nutritional Value 

Over the years, several scientific studies have evaluated and reported the nutritional qualities of different *Dioscorea* species, as shown in Table 1 and Table 2. The nutritional abundance of yam varies depending on the species and variety, as well as the environmental conditions and agricultural practices engaged during planting [35,36]. The analytical method used for estimation also plays a significant role in nutritional levels recorded in yam. The major component of yam is water, which contributes up to 93% of fresh/wet weight of the tuber especially in *D. bulbifera*, *D. delicata* and *D. pentaphylla* [37,38,39]. While the moisture content of other *Dioscorea* species ranges between 51% to 90% (Table 1), it is noteworthy to highlight that *D. hispida* varieties were reported to have the lowest moisture content, ranging from 15.8%–37.8% of fresh weight [32]. High values of moisture have also been reported in other root and tuber crops, with values ranging from 60%–79% in cassava, potato and sweet potatoes [40,41]. The moisture content of roots and tubers plays a very important role in determining the susceptibility of the crops to microbial spoilage and maintaining the shelf life of produce. Thus, species and varieties with low moisture content have longer shelf life and are more suitable for prolonged storage [42,43]. According to FAO, an estimate of about 22% and 39% postharvest losses of yam occur in the major and minor seasons, respectively, due to high moisture content [44], hence contributing significantly to income loss for both farmers and traders. In addition to spoilage caused by high moisture content, it is important to highlight the importance of moisture as it relates to nutritional content of yam. A study on the effect of storage on nutritional content of yam revealed an increase in protein content, total sugar and reducing sugar from 13.0%–14.6%, 6.5%–9.8% and 1.7%–2.3%, respectively, as moisture decreased by 67.8% to 56.5% [45].

### 2.1. Yam as a Source of Dietary Energy

Yam is classified as an energy food source to consumers especially in sub-Saharan Africa (SSA) because of its high starch content which amounts up to 80% in dry weight basis (Table 1) [84]. Among the *Dioscorea* spp., *D. alata* has been reported to contain a relatively high starch content when compared to others, up to 84.3% [54]. A study by Afoakwa et al. [85] evaluated the starch composition of seven of the cultivated yam species (*D. cayenensis*, *D. rotundata*, *D. alata*, *D. bulbifera*, *D. esculenta*, *D. praehensalis*, *D. dumentorum*) in SSA and reported a range between 63.2% and 65.7%. The variation in the starch content of *D. alata* recorded by these authors may be dependent on several environmental factors and agronomic practices, as well as the degree of maturity. The degree of maturity of yam tuber has a great influence on the physicochemical quality of food [86]. In addition, van Eck [87] highlighted the importance of maturity as it influences starch and tuber yield of potatoes when compared to other genetic variations. Similar values of starch have been recorded in other root and tuber crops including potatoes, cassava and cocoyam, as well as cereal grains. While studies have shown high starch content in yams, low (below 1%) starch content were reported in *D. delicata* and *D. olfersiana* (Table 1) [37]. Wang et al. [88] reported a starch content ranging from 20% to 30% for Chinese yam. Yam starch granules consist of a mixture of branched (amylopectin) and un-branched (amylose) chain polymers of D-glucose usually occurring at a percentage ratio of 78:22 [89]; nevertheless the values may vary depending on species as well as genotype. Using the iodo-colorimetric method, Otegbayo et al. [90] reported a wide variability of amylose content between 15.1% and 27.0% of 43 genotypes in 5 species (*D. alata*, *D. rotundata*, *D. cayenensis*, *D. dumetorum* and *D.bulbifera*). Amylose content as high as 39.3 g amylase/100 g starch was reported in Thai yam (*D. hispida*) using a simplified amylose assay [91], comparable to the value reported in Taiwanese *D. alata* (39 g amylose/100 g starch) [92]. A much lower amylose content ranging from 1.4% to 8.7% of starch was recorded for six *D. trifida* of the Venezuelan Amazon [81]. It is important to point out that the discrepancies observed by the latter authors, who reported a wide variability in amylose content of 1.4% to 8.7%, 1.4% to 3.6% and 2.2% to 5.9% was as a result of analytical methods including colorimetric (iodine binding with amylose), differential scanning calorimetry and amperometric, respectively. The ratio of amylose to amylopectin content of yam starches is very crucial as it affects the starch properties and functional characteristics such as crystallinity and digestibility. While high amylopectin content of starch granules results to low levels of retrogradation susceptibility and high peak viscosity, starch granules with high amylose content demonstrates high retrogradation and absorbs limited water content during cooking [93].

In addition, yam contains dietary fiber, which plays a vital role in the digestive system of humans as well as animals. Adequate intake of fiber increases water holding capacity, aids in regular bowel movement, fecal bulkiness and less intestinal transit. It also promotes beneficial physiological effects such as reduction of blood sugar and cholesterol level, trapping of toxic substances and encourages the growth of natural microbial flora in the gut [94,95,96,97]. The crude fiber reported in different species of yam ranged between 0.17% and 18.2% with the minimum and maximum concentrations being recorded in *D. cayenensis* and *D. bulbifera*, respectively. Several dietary fiber constituents such as hemicelluloses, cellulose, lignin and pectins have been reported in yam. Abara et al. [98] examined the dietary fiber components of four raw and cooked *Dioscorea* species (*D. alata*, *D. bulbifera*, *D. cayenensis* and *D. rotundata*) using detergent system analysis and reported low levels of fiber components ranging from 0.08%–0.27% (lignin), 0.80%–1.13% (cellulose) and 0.15%–0.28% (hemicelluloses). Interestingly, no significant difference in dietary fiber was observed between the raw and cooked yams, irrespective of their species. Among the species investigated, *D. bulbifera* had the highest cellulose and hemicelluloses while lignin was higher in *D. alata*. A recent study investigated cell wall carbohydrates of 43 genotypes from five yam species (*D. rotundata*, *D. alata*, *D. bulbifera*, *D. cayenensis* and *D. dumetorum*) using detergent system analysis and recorded the highest cell wall carbohydrate in *D. bulbifera* at 2.1%, 3.2% and 1.1% for hemicelluloses, cellulose and lignin, respectively [99]. The discrepancies of the values in the two studies may be attributed to genotypic variations as highlighted by Otegbayo et al. [99]. In line with this fact, Shajeela et al. [51] observed a higher crude lipid content in *D. oppositifolia* when compared to the other nine *Dioscorea* species investigated. Among the two varieties of *D. oppositifolia* tubers studied by these authors, *D. oppositifolia* var *dukhumensis* (7.42 g/100 g) was reported to contain higher crude lipid than the variety *oppositifolia* (4.40 g/100 g). This trend was also observed for crude protein, with *D. oppositifolia* var *dukhumensis* having higher crude protein content of 13.42 g/100 g compared to 8.44 g/100 g recorded for *D. oppositifolia* var *oppositifolia* [51]. This is in agreement with an earlier study by Arinathan et al. [100]. 

Protein is an essential nutrient required for growth and organ development in humans and animals. It helps in the repair of body tissue, synthesis of enzymes and hormones and also contributes to energy supply. Although roots and tubers are known for their low protein content when compared to pulses/legumes (beans 15%–38%, pea 14%–36%, cowpea 20%–34%, soya bean 29%–50% and groundnut 17%–31%) and cereals such as maize (9.4%), sorghum (11.6%), rice (7.1%), wheat (12.6%) [40,101], yam is reported to have higher dietary protein compared to other root and tuber crops including cassava [102,103]. Yam species like *D. alata* have been reported to have comparable higher protein levels (18.7%) than grains [56]. Contrary to the high protein reported in *D. alata*, Alinnor and Akelezi [78] recorded very low protein content (0.09%) in *D. rotundata* as against 8.28% reported in the same species by another study [77]. Yam tubers are a considerably good source of essential amino acids including phenylalanine and threonine but are limited in tryptophan and sulphur amino acids [102]. In addition, a more recent study on amino acid profiling of different yam species including *D. alata*, *D. bulbifera*, *D. esculenta*, *D. oppositifolia*, *D. pentaphylla*, *D. spicosa*, *D. tomentosa* and *D. wallichi* revealed the prevalence of aspartic acid (5.21–9.36 g/100 g) and glutamic acid (3.20–8.12 g/100 g) in all the yam species investigated [104]. In Africa, consumption of starchy staples, primarily yam and cassava, contributes a great proportion of protein intake in the region ranging from 5.9% in the Southern and Eastern Africa to 15.9% in West Africa [102]. 

Other minor components such as lipids have also been reported in yam. Although these components are present at a very small fraction [103], they have a great impact on the functionality of starch [84]. A wide range of concentrations of lipid between 0.03% and 10.2% have been reported. The highest lipid level (10.2%) was recorded in *D. hamiltonii* [38]. It is important to note that lipid content is highly influenced by the extraction solvent used, as this determines the lipid fraction (bound or unbound) extracted [105]. While lipids supply energy to humans and animals and act as building blocks for cell membranes, they may also serve as pharmacological agents in the body [106]. Mondy and Mueller [107] highlighted the possibility of tuber lipid being of limited nutritional importance; however, it enhances the cellular integrity of the cell membrane, proffers resistance to bruising and reduces enzymatic browning of the tuber.In addition, the ash content of yams is reported to range from 0.1% to 8.8% (Table 1) which is comparable to the values reported in other roots and tubers including potatoes, cassava and cocoyam [108,109,110]. Ash refers to the inorganic residue in any food material and it directly signifies the total amount of minerals present within the food. However, recent studies have shown that ash content measurement of yam starch can be influenced by inefficient starch purification methods, thus leading to higher values. 

### 2.2. Yam as a Source of Minerals

Yams also contain inorganic components such as minerals which play very important roles in the body metabolism (Table 2). These components can be divided into two groups based on their body requirement. They include the macrominerals (potassium, sodium, calcium, phosphorus, magnesium, chloride and sulfur), which are required in larger amounts; and the microminerals or trace minerals including copper, iron, manganese, zinc, iodine, cobalt, fluoride and selenium, needed in the body in small amounts. A study on the mineral profiling of 43 genotypes from five yam species revealed the intra- and inter-species variation in the mineral content of yam [99] as observed for other nutritional components. Potassium, sodium and chloride play a crucial role in the maintenance of total body fluid volume and charge gradients across cell walls [111] and are also responsible for nerve transmission and muscle contraction. The recommended daily allowance (RDA) of potassium in adults and children is 4700 mg/day and 3000 mg/day, respectively. Yams are better sources of potassium than other root and tuber crops (cassava, potatoes and sweet potatoes) as well as cereals (maize, rice, wheat) [112]. Otegbayo et al. [99] reported a range of 775 to 1850 mg/kg of potassium in yam, which correlates with the values previously reported [42,55]. High levels of 1157–2016 mg/100 g dry matter of potassium in different cultivars of *D. alata* was also recorded by a much earlier study [54], thus suggesting that yam contributes immensely to the RDA of potassium for the consumers. 

In contrast to the high potassium concentration recorded in different yam species (Table 2), sodium was detected at lower concentrations (0.35–380 mg/100 g dry weight). Another important macro mineral present in yam is calcium. Calcium is the most abundant mineral in the body with 99% found in bone and teeth, while 1% is found in serum. It plays a vital role in muscle functions, nerve transmission, vascular contraction, intracellular signaling, vasodilation and hormonal secretion [113]. The RDA of calcium (1000–1300 mg) in individuals varies depending on the age, with younger individuals requiring more calcium for the development of bone and teeth [114]. As with other nutrients, calcium content in yam varies with yam species and/or variety. So far, available studies revealed that *D. bulbifera* has a higher calcium content of up to 1410 mg/100 g (Table 2) [66]. It is important to note that metabolism of calcium involves other nutrients such as amino acids and vitamin D, as well as phosphorus. Phosphorus is important in maintaining healthy bones and teeth, acid-base balance of the body and DNA and RNA structure [115]. Phosphorus content was below the RDA (700 mg) recommended for healthy adults in all yam species studied except for *D. remotiflora* (720 mg/100 g) [74]. In addition to the macrominerals mentioned above, yam is reported to be a good source of magnesium. Magnesium plays a vital role in the body metabolic processes, nerve transmission, appropriate muscle tasks and cardiac tempo, as well as synthesis and stability of DNA [116,117]. Shajeela et al. [51] reported magnesium range of 540–634 mg/100 g in two varieties of *D. oppositifolia*, while the authors recorded 532 mg/100 g and 578 mg/100 g of magnesium in *D. pentaphylla* and *D. wallichi*, respectively.

In addition, microminerals such as iron, manganese, zinc and copper have been reported in different yam species, hence, contributing toward the RDA of these nutrients in the body of consumers. Iron is crucial for the formation of hemoglobin in red blood cells that bind and transport oxygen in the body. Bashiri et al. [118] reported the importance of iron in respiration and energy metabolism processes. It also plays a very important role in the immune system and has been implicated in the amalgamation of collagen and neurotransmitters. Otegbayo et al. [99] reported a range between 1.1 and 3.9 mg per 100 g of iron content in different species of yam, with *D. dumetorum* > *D. bulbifera* > *D. alata*> *D. cayenensis* > *D. rotundata*. Although the iron content of different species of yam has been reported to be low as compared to cereals (maize, rice and wheat), Mohan and Kalidass [38] observed very high values of iron (103 mg/100 g) in *D. pentaphylla*. Importantly, the range at which iron is found in a majority of the yam species meets the RDA (11–18 mg/day) of iron. Copper, zinc and manganese are components of numerous enzymes and have also been reported in different species of yam tubers, with values in wild yam being as high as 13.3 mg/100 g (*D. pentaphylla*), 7.1 mg/100 g (*D. remotiflora*) and 9.4 mg/100 g (*D. bulbifera*), respectively [51,74]. While yam contributes immensely to the nutritional requirement of consumers, the non-nutritional components and benefits will be discussed herein.

## 3. Bioactive Compounds in Yam

In addition to the nutritional constituents of *Dioscorea* species, few studies have explored the pharmaceutical potentials of different species of *Dioscorea*. They contain substantial amounts of secondary metabolites referred to as bioactive compounds. Bioactive compounds are produced within the plants besides the primary biosynthetic and metabolic routes associated with plant growth and development. These compounds are not needed for their daily functioning but may provide various functions such as protection, attraction or signaling to the plant [119]. Bioactive compounds can be described as phytochemicals found in plants/food that have the capacity to influence the cellular or physiological activities in humans as well as in animals. They modulate metabolic processes by exhibiting numerous beneficial health effects such as anti-oxidative, anti-hypertensive, anti-inflammatory and anti-diabetic activities, inhibition of receptor activities, inhibition or induction of enzymes and induction and inhibition of gene expression, thus resulting in the promotion of better health [120]. However, it is worth noting that bioactive compounds can also have antinutritional properties, thus eliciting toxicological effects in humans and animals. A wide list of bioactive compounds such as phenolics, flavonoids, allantoin, dioscin, dioscorin, diosgenin, polyphenols, tannins, hydrogen cyanide, oxalate, saponin and alkaloids have been reported in yam by several studies as listed in Table 3. Their content in yam varies within and between species as reported by Wu et al. [35]. These authors reported a varied range of 0.032%–0.092% dry weight and 0.62%–1.49% dry weight of dioscin and allantoin detected in 25 yam landraces from four species (*D. alata*, *D. polystachya*, *D. persimilis* and *D. fordii*), respectively. Inter- and intra-species diversity as it relates to bioactive compounds content has also been highlighted by Price et al. [121]. Using high performance liquid chromatography (HPLC), dioscin ranging between 0.086 and 0.945 µg/mLwas reported in yams of African origin including *D. cayenensis*, *D. mangenotiana* and *D. rotundata*. In addition, dioscin has been reported in other parts of yam plant such as rhizomes and roots as reviewed by Yang et al. [122]. Hence, this section will elucidate the bioactive constituents of yams.

### 3.1. Steroidal Saponin

Saponins are a diverse group of glycosidic compounds containing triterpenoid and steroidal aglycone that occur naturally in plants and in lower marine organisms. While triterpenoid saponins are mostly found in dicotyledonous angiosperms, steroidal saponins are mainly present in monocot species such as *Dioscoreaceae* [134]. Steroidal saponins vary in their structural constitutive frameworks, sugars and aglycones, leading to a broad range of biological activities exerted by these compounds. Depending on the aglycone moiety, steroid saponins can be classified into spirostane, stigmastane, furostane, cholestane, ergostane and pregnane families [135]. Members of the *Dioscorea* genus mostly contain spirostane and furostane steroid glycosides; however, studies have reported the possible presence of other steroidal saponins [136,137]. Predominantly, yams contain a spirostane steroidal sapogenin known as diosgenin with a structure of 25R-spirost-5-en-3b-ol consisting of a hydrophilic sugar moiety linked to a hydrophobic steroid aglycone with a molecular formula C_27_H_42_O_3_ [138,139]. 

The presence of diosgenin, an aglycon of dioscin has also been reported in several species of yams, making yam one of the leading sources of steroidal sapogenin diosgenin, a precursor used for the synthesis of the steroidal drugs estrogen and progesterone in the pharmaceutical industry [140]. The synthesis of cortisone and hormonal drugs such as sex hormone, progestational hormone and other steroids with diosgenin extracted from *D. zingiberensis*, *D. villosa* and *D. composite* have been reported [141,142]. The utilization of diosgenin is linked to its pharmacological activities and medicinal properties including decreasing oxidative stress, inducing apoptosis, suppressing malignant transformation, preventing inflammatory events, promoting cellular differentiation/proliferation and regulating the T-cell immune response, thus, resulting in antidiabetes, anticancer, neuro- and cardiovascular protective, immunomodulatory, estrogenic and skin protective effects [134,138,143,144]. A study by Tada et al. [145] examined the efficacy of diosgenin extracted from *D. composita* or *D. villosa* against skin aging. Their findings revealed potential of diosgenin to enhance DNA synthesis of skin using a human 3D skin equivalent model anda restoration of keratinocyte proliferation in aged skin. In addition, spirostanes possess better antimicrobial activity when compared to other steroid glycosides; however, the activity is dependent on the type and sequence of the sugars [135].

The underlying mechanism of action of diosgenin may vary depending on the disease and has been reviewed by Cai et al. [139], Chen et al. [143] and Raju and Rao [138]. Although diosgenin has been reported in several yam species, its content varies considerably within the *Dioscorea* genus with *D. barbasco* (Mexican wild yam) and *D. zingiberensis* (Chinese yam) being very important sources for diosgenin [146,147]. Perhaps this explains the reason why China and Mexico account for more than half (about 67%) of world diosgenin production [148] and the utilization of this compound takes preeminence in these countries. The presence of diosgenin has also been recorded in *D. alata* [144]. Contreras-Pacheco et al. [149] use gas chromatography-mass spectrometry (GC-MS) to quantify and characterize diosgenin in sixty accessions of two *Dioscorea* species (*D. sparsiflora* and *D. remotiflora*) collected from the city of Jalisco, Mexico. Their findings showed diosgenin at a range between 0.02 and 0.16 mg/kg in dry basis. In the same vein, Yi et al. [146] recorded a range of 0.78 mg/g to 19.52 mg/g in three *Dioscorea* species including *D. zingiberensis*, *D. septemloba* and *D. colletti*; however, diosgenin was not detected in *D. polystachya*. Huai et al. [150] revealed that the intra-species diversity with respect to significant differences in the amount of disogenin in different yam varieties may be attributed to climatic factors and environmental conditions such as growing and storage conditions. 

In addition to diosgenin, other steroidal saponins have been reported in several *Dioscorea* species. An extensive review by Sautour et al. [151] revealed over 50 saponins in 13 *Dioscorea* species, including *D. cayenensis*, *D. bulbifera*, *D. colletii*, *D. futschauensis*, *D. deltoidea*, *D. panthaica*, *D. nipponica*, *D. pseudojaponica*, *D. parviflora*, *D. spongiosa*, *D. polygonoides*, *D. zingiberensis* and *D. villosa*. The authors also highlighted the pharmacological properties of the saponins as regards cytotoxic and antifungal properties.

### 3.2. Dioscorin

Dioscorin is the main storage protein of the Dioscorea tuber, accounting for approximately 90% of extractable water-soluble proteins [152,153]. Yam tubers contain two dioscorin proteins, dioscorin A and dioscorin B, encoded by genes that share about 69% sequence similarity [154,155]. Using Raman spectroscopy, Liao et al. [155] showed that the secondary structure of dioscorin A (molecular weight [MW] ~ 33 kDa) of *D. alata* L. is mostly of alpha-helix whereas that of dioscorin B (MW ~ 31 kDa) belongs to anti-parallel β-sheet. The authors also highlighted that the major amino acids (phenylalanine, tyrosine, methionine, tryptophan and cysteine) microenvironment exhibited a clear difference between dioscorin A and B [155]. An earlier study by this group of authors on the secondary structure of dioscorin of three yam species (*D. alata* L., *D. alata* L. var. *purpurea* and *D. japonica*) reported the similarity in molecular mass across the three species. However, they observed dissimilarity in the amino acid composition and secondary structure of dioscorin between *D. alata* L., *D. alata* L. var. *purpurea* and *D. japonica* [156]. 

In contrast to other storage proteins, dioscorin also exhibits enzymatic activities, such as carbonic anhydrase, trypsin inhibitor, dehydroascorbate reductase, monodehydroascorbate reductase and lectin activities [152,157,158,159]. The antioxidant potential of dioscorin purified from yam tuber has also been reported [160,161]. Liu et al. [161] examined the antioxidant activities of dioscorin in the tubers of two *Dioscorea* species (*D. alata L*. and *D. batatas* [presently known as *D. polystachya*]) using (2,2-diphenyl-1-picryl-hydrazyl-hydrate) and hydroxyl radicals scavenging activity assay, reducing power test and anti-lipid peroxidation test. Their findings revealed that dioscorins from the two yam species exhibited different scavenging activities against DPPH (1, 1-diphenyl-2-picrylhydrazyl) and hydroxyl radicals, with *D. alata* dioscorin showing higher antioxidant and scavenging activities as compared to that of *D. polystachya*. This variation is ascribed to the variation in amino acid composition and protein conformations [161]. Dioscorin has been reported to inhibit the activities of angiotensin converting enzymes, thus suggesting its potential for control of hypertension [162]. In addition, Fu et al. [163] demonstrated the potential of dioscorin isolated from *D. alata* as a Toll-like receptor 4 (TLR4) activator as well as an inducer of the cytokine expression in macrophage through TLR4-signalling pathways, thus, resulting in the activation of innate and adaptive immune system. 

### 3.3. Alkaloids 

Alkaloids are a large and structurally diverse group of amino acid-derived heterocyclic nitrogen compounds of low molecular weight, widely distributed across plant kingdoms, microorganisms and animals and deriving their name from their alkaline chemical nature [164]. Due to the complexity of alkaloids, no single taxonomic principle could completely classify them [165]. Alkaloids can be grouped into classes based on their natural and biochemical origin, as well as by chemical structures (heterocyclic and non-heterocyclic alkanoids). Structurally, they can be divided into classes such as quinolines, isoquinolines, indoles, pyrrolidines, pyridines, pyrrolizidines, tropanes and terpenoids and steroids [166]. Presently, over 18,000 alkaloids have been reported in different plant species [167] including those of *Dioscorea*. While alkaloids have been utilized pharmaceutically because of their therapeutic activities such as anti-microbial, anti-hypertensive, anti-cancer, anti-inflammatory, anti-human immunodeficiency virus (HIV) and many others, some alkaloids are highly toxic to humans and animals [164,165,168]. They may contribute to undesirable sensory qualities such as bitterness in food crops such as yams [21]. Alkaloids have been reported in several species of yams (*D. alata*, *D. oppositifolia*, *D. hamiltonii*, *D. bulbifera*, *D. pubera*, *D. pentaphylla*, *D. wallichii*, *D. glabra* and *D. hispida*) at values between 7.2 and 16 mg per 100 g dry weight [123] (Table 3). A study from West Africa characterized the antinutritional factors in flour samples from four *Dioscorea* species and reported alkaloid levels ranging between 0.02 and 0.11 mg/100 g [169]. Similarly, Senanyake et al. [170] recorded alkaloid levels of 0.94, 1.64 and 1.89 mg/100 g in *D. alata* (Rajala), *D. alata* (Hingurala) and *D. esculenta* (Kukulala), respectively. Alkaloid has also been reported in *D. belophylla* (Prain) Haines at a concentration of 0.68 mg/100 g [125]. 

One of the major alkaloids in yam is dioscorine, a toxic isoquinuclidine alkaloid with molecular formula C_13_H_19_O_2_N [171,172]. Dioscorine has been reported in several yam species including *D. hispida*, *D. hirsute*, *D. dumetorum* and *D. sansibarensis* (Table 3) [128]. The presence of dioscorine in yam is associated with bitter taste and has been shown to induce nausea, dizziness and vomiting. Dioscorine has exhibited the potency to trigger fatal paralysis of the central nervous system when ingested [4], a reason that explains the use of dioscorine in the production of poisons for hunting purposes. Due to the water solubility of this toxin, it is easily removed by the traditional processing methods used for yam processing such as washing, boiling and soaking.

### 3.4. Flavonoids

Flavonoids, ubiquitous in photosynthesizing cells, naturally occur as aglycons, glycosides and methylated derivatives [173]. Structurally, flavonoids (C_6_-C_3_-C_6_) contain a 2-phenyl-benzo(α)pyrane or flavane nucleus, which comprises two benzene rings (A and B) linked through a heterocyclic pyrane ring (C) [174]. Based on the position of the carbon of the C ring (on which B ring is attached), the degree of unsaturation as well as oxidation of the C ring, flavonoids can be classified into subgroups [175]. For isoflavones, the B ring is linked in position 3 of the C ring, while the B ring of neoflavonoids is linked to position 4 of the C ring. Other subgroups of flavonoids in which the B ring are linked to position 2 include chalcones, flavones, flavonols, catechins, flavanonols, flavanones and anthocyanins. The pharmacological potential of these compounds cannot be overemphasized. Flavonoids have shown antioxidant, anti-inflammatory, antihypertensive, antidiabetic, antimicrobial, anticonvulsant, sedative, antidepressant, anti-proliferative, anticancer, cardioprotective, antiulcerogenic and hepatoprotective activity [176]. 

The presence of flavonoids has been reported in wide varieties of yams (Table 3). A recent study by Padhan et al. [177] investigated the flavonoid content of nine *Dioscorea* species including *D. alata*, *D. oppositifolia*, *D. hamiltonii*, *D. bulbifera*, *D. pubera*, *D. pentaphylla*, *D. wallichii*, *D. glabra* and *D. hispida*. Their findings revealed flavonoid content ranging from 0.62 to 0.85 mg/g dry weight, of which levels detected in *D. alata* and *D. hispida* were significantly lower compared to other *Dioscorea* species. In addition, the authors reported potential antioxidant activities of the yam tuber extracts to range from 1.63 to 5.59%. *D. bulbifera* and *D. pubera* with significantly higher amount of bioactive compounds such as flavonoids exhibited higher radical scavenging activity compared to other *Dioscorea* species irrespective of the screening method (DPPH, ABTS, nitric oxide and superoxide radical scavenging assay) used [177]. Flavonoids have also be quantified in *D. belophylla* (Prain) Haines (8.8 mg/100 g), *D. alata* (Rajala) (5.2 mg/100 g), *D. alata* (Hingurala) (9.8 mg/100 g) and *D. esculenta* (Kukulala) (12.4 mg/100 g) [125,170]. Another Nigerian study also reported flavonoid content as well as the associated antioxidant activity of three yam species (*D. cayenensis*, *D. dumetorum* and *D. bulbifera*) [60].

### 3.5. Phenols and Phenolic Acids

Phenols and phenolic acids are a group of abundant secondary metabolites found in plants. Simple phenol is characterized by one or more hydroxyl groups (-OH) attached directly to the aromatic system and comprising of resorcinol, phenol, phloroglucinol and catechol [178,179]. On the other hand, phenolic acids are used to describe phenolic compounds having a benzene ring, a carboxylic group and one or more hydroxyl and/or methoxyl groups in the molecule [180]. Phenolic acids are rarely present in free form, occurring in bound form such as esters, amides or glycosides [181]. They comprise two parent structures, the hydroxybenzoic acid and hydroxycinnamic acid. While the hydroxybenzoic acid (vanillic, gallic, protocatechuic and syringic acid) are the simplest phenolic acids found in nature consisting of seven carbon atoms (C_6_-C_1_), hydroxylcinnamic acids (ferulic, caffeic, sinapic and *p*-coumaric acid) are the most common in fruits and vegetables and have nine carbon atoms (C_6_-C_3_) [182].

*Dioscorea* species have been identified as a possible source of phenols as well as phenolic acids (Table 3). Zhao et al. [183] evaluated the total phenolic acids of two yam species (*D. oppositifolia* and *D. hamiltonii*) using an HPLC system. Their findings reported the presence of total phenolic acid in both yams; however, the content in *D. oppositifolia* (297.3 mg/mL) was almost double that of *D. hamiltonii* (158.2 mg/mL) which contributed to the significantly better antioxidant, anti-inflammatory and immune regulation effects of *D. oppositifolia* compared to *D. hamiltonii*. Among the phenolic acids detected in the two yam species, syringic acid was recorded the highest in both yams [183]. Similarly, a study profiled the phenolic compounds in *D. alata* andreported the presence of ferulic, sinapic, caffeic and *p*-coumaric acid and vanillic acid [184]. An earlier study reported phenolic constituents in 10 yam cultivars from five species highlighting the prevalence of these compounds in *D. alata* and *D. bulbifera* when compared to other species (*D. cayenensis*, *D. dumetorum* and *D. rotundata*) irrespective of the cultivar [185]. The phenolic concentration of *D. rotundata* (12–69 mg catechin/100g) was the lowest among the five species and Graham-Acquaah et al. [186] reported a similar range (20–37 mg catechol/100 g) in two cultivars of *D. rotundata.* The latter authors observed a significant variation across tuber sections. In one *D. rotundata* cultivar (*Puna*), the order of concentration of phenol in the sections were head > mid-section > tail, whereas the head and mid-section of *Bayere fitaa* cultivar had a similar phenol concentration but significantly higher than that of the tail section [186]. Similarly, Padhan et al. [177] found significant variation in the phenol content (2.1–9.62 mg/g dry weight) of various yam species (*D. alata*, *D. bulbifera*, *D. oppositifolia*, *D. pubera*, *D. hamiltonii*, *D. pentaphylla*, *D. glabra*, *D. hispida* and *D. wallichii*), with a significantly higher concentration in *D. bulbifera* compared to other species.

### 3.6. Other Bioactive Compounds

In addition to the bioactive compounds described above, tannins, phytates and oxalates have been reported in different species of yam (Table 3) [42,54,99,187]. Their content in yams varies depending on species, variety, soil type and other environmental factors. The presence of tannin, phytate and oxalate ranging from 56–1970 mg/kg, 270.7–379.4 mg/kg and 487–671 mg/kg on a dry matter basis, respectively, were recorded in 43 genotypes from five yam species (*D. alata*, *D. rotundata*, *D. dumetorum*, *D. bulbifera* and *D. cayenensis*) of major landraces in Nigeria [99]. These compounds are referred to as anti-nutritional compounds because of the toxic effects associated with their consumption. Tannins are water-soluble polyphenols known for their astringent taste and ability to bind to and precipitate various organic compounds including proteins, amino acids and alkanoids, thus decreasing digestibility and tastiness [188]. Structurally, tannins are classified into two groups, the hydrolysable and the condensed tannins. Studies on experimental animals showed possible effects of tannins on feed intake and efficiency, net metabolizable energy, growth rate and protein digestibility [189]. Their relationship with reduced sensory quality of food cannot be neglected. Other adverse effects of tannins such as increase in excretion of protein and essential amino acids and damage to the mucosal lining of the gastrointestinal tract have also been reported [189]. On the other hand, studies have also shown the pharmacological potential of tannins including antioxidant and free radical scavenging activity; anticarcinogenic, antimutagenic, cardio-protective properties; and antimicrobial activities [190]. 

Phytate (*myo*-inositol hexa*kis*phosphate), a salt form of phytic acid, is the major storage form of phosphate and inositol found in a wide range of plants [191]. Its classification as an antinutrient is associated with its capacity to form complexes with nutrients especially dietary minerals including zinc, calcium and iron, thus reducing their availability in the body and causing mineral related deficiency in humans. In addition, the formation of insoluble complexes by phytate with other food components such as protein, lipids and carbohydrate have been reported thereby negatively impacting the utilization of these nutrients [191,192]. Notwithstanding these negative effects, dietary phytate exerts numerous positive health effects on humans including anticancer and antidiabetes activities and protection against renal lithiasis, dental caries, HIV and heart related diseases as extensively reviewed by Kumar et al. [191]. On the other hand, oxalate, salt of oxalic acid, occurs as an end product of metabolic processes in plant tissues. Oxalates may occur as insoluble calcium oxalate, soluble oxalate or in combination of the two forms as reported for yam tubers [99]. They bind to minerals especially calcium, magnesium and iron, resulting in unavailability of these minerals to human and animal consumers [193]. Other detrimental effects such as intense skin irritation as a result of contact with *Dioscorea* mucilage has been linked to the presence of calcium oxalate crystals.

Furthermore, hydrogen cyanide which is formed as a result of hydrolysis of glycosides by enzymes in plants and is a neurotoxin found in cassava (*Manihot esculenta*), has been reported in yam though at lower concentrations. Shajeela et al. [51] reported hydrogen cyanide ranging from 0.16 to 0.34 mg per 100 g in nine *Dioscorea* species with the highest level recorded in *D. tomentosa* and *D. oppositifolia* var *oppositifolia.* Using spectrophotometry methods, cyanide was also reported in *D. alata* and *D. hispida* Dennst sampled from Sleman, Yogyakarta [194]. Albeit of the antinutritional properties of yam tubers, steps and methods of processing before consumption have proven to efficiently destroy these toxic compounds [99,195].

## 4. Therapeutic Potentials of Yams

Pharmaceutical and phytomedical products derived from plants have a long history of use by natives as traditional medicine and a proven evidence of efficacy. Gurib-Fakim [196] highlighted that tribal people in the tropics use plants for medicine as direct therapeutic agents and starting points for the elaboration of semi-synthetic compounds. A majority of secondary plant compounds used in modern medicine were identified through ethnobotanical investigations. Ethnobotany is an interdisciplinary field of research with specific focus on the empirical knowledge of indigenous people with respect to natural plant substances that influence health and wellbeing and their associated risk [196]. Natives of different ethnic communities that either cultivate or have wild *Dioscorea* spp. have utilized them for medicinal purposes (Table 4). Unfortunately, documentation of the importance and utilization on *Dioscorea* is still limited. Research has shown that yam bioactive compounds and its supplementations play vital roles in weight changes, activities of carbohydrate digestive and transport enzymes, changes in the morphology of intestines, alterations in blood lipids, lipid peroxidation reduction and liver damage prevention [197]. A recent study by Pinzon-Rico and Raz [198] highlighted the high demand and robust market of four wild yam species including *D. coriacea*, *D. lehmannii*, *D. meridensis* and *D. polygonoides* in Bogota, Colombia. The four species have been implicated in blood purification probably because of their effect on reducing blood cholesterol, triglycerides, uric acid and glucose. The general acceptability and long history of local consumption of yams among various communities across the continents may be attributed to its safety and portends high regulatory acceptability [199]. Current research has shown that yams contain substantial amounts of secondary metabolites referred to as bioactive compounds that have pharmaceutical potentials as discussed and the health benefits associated with yam consumption is discussed hereafter.

### 4.1. Antimicrobial Potential of Yam

Over the years human medicine has improved greatly; but infections caused by microbes such as bacteria, viruses, fungi and parasites remain a lingering hurdle to overcome, especially with the emergence of widespread drug resistant forms of these microbes and adverse side effects to certain antibiotics [263]. Research into plant sourced antibiotics has intensified and the antimicrobial potentials of certain yam species have been investigated and reported. Using crude extracts and compounds isolated from the bulbils of the African medicinal plant *D. bulbifera,* Kuete et al. [264] showed that these extracts and compounds can be effective drugs against a wide range of resistant gram negative bacteria. The inhibitory effect of the extracts was dependent on the concentration but still less effective compared to standard antibiotics. Likewise tuber mucilage extract of *D. esculenta* have exhibited antibacterial properties against three human bacterial strains including *Escherichia coli*, *Pseudomonas aeruginosa* and *Staphylococcus aureus* [263]. The inhibitory potential of *D. alata* tuber extracts against *Salmonella typhimurium*, *Vibrio cholerae*, *Shiegella flexneri*, *Streptococcus mutans* and *Streptococcus pyogenes* have also been reported [31]. In addition, endophytic fungi isolated from rhizome extract of *D. zingiberensis*, a Chinese medicinal plant, has shown its antibacterial potential for use for the production of antibacterial natural products [265]. The same trend was observed by Sonibare and Abegunde [127]. Using the agar well diffusion and pour plate method, the authors reported extracts of *D. dumetorum* and *D. hirtiflora* tubers as possible sources of antimicrobial agents with their antimicrobial efficacy directly linked to the phenolic contents of the plants and DPPH scavenging activity. Kumar et al. [24] compared the antibacterial activity of *D. pentaphylla* tuber extracts and antibiotics (penicillin and kanamycin) on five selected bacterial strains (*Vibrio cholera*, *Shigella flexneri*, *Salmonella typhi*, *Streptococcus mutans* and *Streptococcus pyogenes*). Their findings revealed a significant inhibitory activity of *D. pentaphylla* tuber extracts against the tested bacteria. This activity was attributed to diosgenin content in the tubers.

### 4.2. Antioxidant Activities of Yam

Antioxidant activities have been reported in different species of *Dioscorea*, including *D. alata*, *D. bulbifera*, *D. esculenta*, *D. oppositifolia* and *D. hispida* (Table 4) [266,267,268,269,270]. Using a DPPH assay, Murugan and Mohan [268] reported radical scavenging activity of 79.3% for 1000 µg/mL *D. esculenta* extract with IC_50_ value of 38.33 µg/mL, whereas IC_50_ value of 18.25 µg/mL was recorded for the reference standard (ascorbic acid). The same trend was observed by the author when the ABTS assay was used, with radical cation scavenging activity range of 46.1% to 64.1% at concentration between 125 and 1000 µg/mL and IC_50_ value of 40.50 µg/mLwhile IC_50_ value was 20.67 µg/mLfor trolox. The author attributed the antioxidant and free radical scavenging activity to high content of total phenolic and flavonoid compounds. Similarly, Padhan et al. [177] examined the antioxidant activity of nine different yams (*D. alata*, *D. bulbifera*, *D. pentaphylla*, *D. pubera*, *D. glabra*, *D. oppositifolia*, *D. wallichii*, *D. hispida* and *D. hamiltonii*) cultivated in Koraput, India. Their findings revealed antioxidant capacity ranging from 1.63% to 5.59%, with IC_50_ values of 101–1032, 77.9–1164, 47–690 and 27–1023 µg/mLfor ABTS, DPPH, nitric oxide and superoxide scavenging activity, respectively. Among the yam species evaluated, antioxidant capacities of *D. pubera*, *D. pentaphylla* and *D. bulbifera* were significantly higher with lower IC_50_ values than the standards when compared to the other species. The variation in scavenging activities observed in the different yam species is attributed to the disparity in the content of the bioactive compounds in the yam species [177]. 

### 4.3. Anti-Inflammatory Activity of Yam

Several animal studies have reported the anti-inflammatory activity of *Dioscorea* species. Olayemi and Ajaiyeoba [271] investigated the anti-inflammatory potential of defatted methanol extract of *D. esculenta* tuber on Wistar rats. Their finding showed a significant dose-dependent inhibition of the carrageenan at doses of 100 mg/kg and 150 mg/kg which was comparable to that of 150 mg/kg acetylsalicylic acid (reference standard). Chiu et al. [130] confirmed that *D. japonica* ethanol extract elicited an in vivo anti-inflammatory effect on mouse paw oedema induced by λ-carrageenan. Pre-treatment using dried yam (*Dioscorea* spp.) powder on Sprague-Dawley rats before inducement of duodenal ulcer by intragastric administration of cysteamine-HCl (500 mg/kg) revealed that dried yam powder exerted a significant protective effect by reducing the incidence of perforation caused by cysteamine and preventing duodenal ulcer, which was comparable to the pantoprazole effect [272]. The observed effect of yam powder was attributed to its potential to lower inflammatory cytokines as well as scavenging free radicals and up-regulating activity of carbonic anhydrase. The hydro-methanol extract of *D. alata* tubers which contain different bioactive phytocompound has also shown to significantly down-regulate the pro-inflammatory signals in a gradual manner compared to a reference control (µg/mL) [203]. Mollica et al. [273] reported the anti-inflammatory activity of extract from *D. trifida* on food allergy induced by ovalbumin in mice. In addition extracts from leaf, rhizome and bulbil have exhibited anti-inflammatory activity.

### 4.4. Anticancer Activity of Yam

Synthetic medications and chemotherapy for cancer management comes with a multitude of side effects that are often intolerable for most cancer patients; thus, naturally occurring bioactive compounds in plants are increasingly becoming better alternatives.In vitrocytotoxicity screening provides insights and preliminary data that help select plant extracts with potential anticancer properties for future work andin vivoreplication. A study by Itharat et al. [240] showed that aqueous and ethanol extracts of rhizome of *D. membranacea* and *D. birmanica* were cytotoxic against three human cancer cell lines while remaining non cytotoxic to normal cells. The use of active compounds naphthofuranoxepins (dioscorealide A and B) and dihydrophenanthrene from *D. membranacea* (locally known as Hua-Khao-yen) rhizome in Thai medicine is highly potent and has exhibited cytotoxic activity against five types of human cancer cells [274,275,276]. This was supported by a more recent study, which highlighted the utilization of dioscorealide B as a possible anticancer agent for liver cancer and cholangiocarcinoma [277]. The hepatotoxic compound diosbulbin B has also been reported as a major antitumor bioactive component of *D. bulbifera* (air potato) in dose-dependent manner, with no significant toxicity in vivo at dosage between 2 and 16 mg/kg [278,279]. 

Plants with steroidal saponins have exhibited anticancer effects [280,281,282] and these bioactive compounds are abundant in different *Dioscorea* species. According to Zhang et al. [283], deltonin exerts an apoptosis-inducing effect, which may correlate with ROS-mediated mitochondrial dysfunction, as well as the activation of the ERK/AKT signaling pathways, thereby suggesting deltonin as a potential cancer preventive and therapeutic agent [284]. Cytotoxicity studies using steroidal saponins from *Dioscorea collettii* var. *hypoglauca* showed they were active against human acute myeloid leukemia under in vitroconditions [285]. In an anticancer drug screen by the National Cancer Institute (NCI), USA, protoneodioscin, a furostanol saponin compound isolated from *Dioscorea collettii* var. *hypoglauca*, exhibited cytotoxicity effects against most cell lines including leukemia, central nervous system, colon, prostate cancer [227]. It is interesting to note that no compound in the NCI data base shares a similar cytotoxicity pattern to those of protoneodioscin, thus indicating a unique anticancer pathway. The polysaccharide of RDPS-I purified from the water extract of Chinese yam tuber exerted a significant inhibition on the cancer cell line of melanoma B16 and Lewis lung cancer in mice in-vivo [286]. Another study by Chan and Ng [279] investigated the biological activities of lectin purified from *D. polystachya* cv. Nagaimo. The authors observed after 24 h treatment the inhibitory role of lectin on the growth of some cancer cell lines including nasopharyngeal carcinoma CNE2 cells, hepatoma HepG2 cells and breast cancer MCF7 cells, with IC50 values of 19.79 μM, 7.12 μM and 3.71 μM, respectively. Through the induction of phosphatidylserine externalization and mitochondrial depolarization, it has been revealed that *D. polystachya* lectin can evoke apoptosis in MCF7 cells [279]. Furthermore, diosgenin has been reported to significantly inhibit the growth of sarcoma-180 tumor cellsin vivo while enhancing the phagocytic capability of macrophages in vitro, thus suggesting that diosgenin has the potential to improve specific and non- specific cellular immune responses [287]. The anticancer mechanism of action for diosgenin may be attributed to modulation of multiple cell signaling events including molecular candidates associated with growth, differentiation, oncogenesis and apoptosis [288]. 

### 4.5. Anti-Diabetic Activity of Yam 

Notwithstanding the availability of numerous anti-diabetic medicines in the pharmaceutical industry and market, diabetes and related complications remain a medical burden. Plants’ anti-diabetic potential stems from their ability to restore the function of the pancreatic tissues which leads to three possible outcomes: increasing the insulin output, inhibiting the intestinal absorption of glucose and restoring the facilitation of metabolites in insulin dependent processes [234]. There is minimal evidence on specific action pathways in the treatment of diabetes; however, we can infer that most plants that contain bioactive substances such as flavonoids, alkaloids and glycosides offer a buffer to patient management [289]. *D. dumetorum*, commonly known as bitter yam, has long been proven to play active role in the treatment of diabetes in traditional medicine due to its hypoglycemic effect [233]. Literature reveals that aqueous extract of *D. dumetorum* tuber, known for its alkaloid (dioscoretine) content, control hypercholesterolemia, hyperlipidemia and hyperketonemia [234]. In 2015, a study which evaluated the anti-diabetic potential and free radical scavenging activity of copper nanoparticles (CuNPs) synthesize with the aid of *D. bulbifera* tuber extract revealed a promising antidiabetic and antioxidant properties [210]. In animal studies, extract of *D. bulbifera* and *D. alata* tuber showed significant reduction in blood glucose level as well as increased body weight in rats treated with streptozotocin and alloxan, respectively [290,291]. Another study showed, however, consumption of *D. bulbifera* by female diabetic rats decreased hyperglycemia and bone fragility [292]. A similar trend was observed on dexamethasone-induced diabetic rats treated with *D. polystachya* extract [293].

The quest for novel drugs in the clinical treatment of diabetic complications such as peripheral neuropathy has led to the discovery of DA-9801, an ethanol extract of *D. japonica*, *D. rhizoma* and *D. nipponica*, as a potential therapeutic agent [294,295]. Peripheral neuropathy is a common disorder among diabetic patients, a result of the malfunctioning of the peripheral nerves. Peripheral neuropathy is characterized by symptoms such as pain, numbness and chronic aberrant sensations, which often disrupt sleep and can lead to depression, thus affecting the quality of life [238]. An investigation conducted by Song et al. [296] on the inhibitory effects of DA-9801 on transport activities of clinically important transporters showed that inhibitory effects in vitro did not translate into in vivo herb drug interaction in rats. Interestingly, Jin et al. [297] and Moon et al. [238] further buttressed the potential therapeutic applications of DA-9801 for the treatment of diabetic peripheral neuropathy. These studies show that DA-9801 reduced blood glucose levels and increased the response latency to noxious thermal stimuli. It is anticipated that DA-9801 can be used as a botanical drug for the treatment of diabetic neuropathy. Transporters are critical in the absorption, distribution and elimination of drugs, thus modulating efficacy and toxicity [296]. This prediction of interaction is vital in clinical studies and the drug development process. Sato et al. [298] demonstrated that the natural product diosgenin remains a candidate for use in acute improvement of blood glucose level in type I diabetes mellitus. Also, Omoruyi [299] supports the use of *D. polygonoides* extracts in clinical management of metabolic disorders such as diabetes.

### 4.6. Anti-Obesity and -Hypercholesterolemic Activities of Yam 

Jeong et al. [300] reported the anti-obesity effect of *D. oppositifolia* extract on diet-induced obese mice. In their study, a high-fat diet was given to female mice with 100 mg/kg of *n*-butanol extract of *D. oppositifolia* for 8 weeks. The authors observed a significant decrease in total body weight and parametrial adipose tissue weight; as well as decrease in total cholesterol, triglyceride level and low density lipoprotein (LDL)-cholesterol in blood serum; female mice associated with the ingestion of *D. oppositifolia n*-butanol extract. The observed effect of *D. oppositifolia n*-butanol extract is mediated through suppression of feeding efficiency and absorption of dietary fat [300]. An earlier study, which evaluated the anti-obesity effect of methanol extract of *D. nipponica* Makino powder, reported the effectiveness of the extract against body and adipose tissue weight gains in rodents induced by a high-fat diet [301]. The anti-obesity potential of extract of *D. steriscus* tubers extracted using a solvent cold percolation method have been reported [302]. When compared with a commercially available anti-obesity medication (herbex), *D. steriscus* tubers extract showed a significantly higher anti-obesity activity. The author attributed the result to be associated with the bioactive compounds of *D. steriscus* tubers, which can act as lipase and α-amylase inhibitors and thus are useful for the development of anti-obesity therapeuticals [302].

Extracts of *Dioscorea* species have been used in clinical management of other metabolic disorders such as abnormal cholesterol level. Several animal studies have shown the antilipemic effects of sapogenin and diosgenin-rich extract of *Dioscorea* species like *D. polygonoides* (Jamaican bitter yam) on hypercholesterolemic animals such as mice and rat, thus resulting in the reduction in the concentrations of blood cholesterol [303]. Another study which investigated the effect of *D. alata* L. on the mucosal enzyme activities in the small intestine and lipid metabolism of adult Balb/c mice showed constant improvement in the cholesterol profile of the liver and plasma of mice fed with 50% raw lyophilized yam for a duration of 21 days [304]. The authors also observed an increase in fecal excretions of neutral steroid and bile acids whereas absorption of fat was reduced in mice fed with 50% yam diet. Yeh et al. [305] observed a significant reduction in plasma triglyceride and cholesterol in male Wistar rat as a result of consumption of a 10% high cholesterol diet supplemented with 40% *D. alata*.

### 4.7. Yam as an Agent for Degenerative Disease Management

In an animal study using Swiss albino mice with streptozotocin induced dementia, *D. bulbifera* tubers were reported as having the potential to preserve memory while serving as a preserving, curing and restorative agent [209]. The authors further highlighted the possible delay of onset of neurodegenerative diseases as well as mitigation with the ingestion of dietary polyphenols that confer protection to oxidative stress and neurodegeneration. Also, the neuroprotective effect of *D. pseudojaponica Yamamoto* using senescent mice induced by D-galactose indicated the useful potential of yam for treatment of cognitive impairment, a process partly mediated via enhancing endogenous antioxidant enzymatic activities [306]. The steroidal saponin—diosgenin—one of the major bioactive compounds in yam, was found to aid the restoration of axonal atrophy and synaptic degeneration, thus improving memory dysfunction in transgenic mouse models of Alzheimer’s disease [307]. Diosgenin administration prior to surgery in rat test models reduced significantly the death rate while improving impaired neurological functions, thus establishing the potential cerebral protection of diosgenin against transient focal cerebral ischemia- reperfusion (I/R) injury [254]. In an in vivo study using mice, the same group of authors reported that diosgenin enhanced neuronal excitation and memory function in normal mice, which is mediated by 1,25D3-MARRS (membrane associated, rapid response steroid-binding) triggered axonal growth [308]. This seems to support a school of thought that sees diosgenin as a new category of cognitive enhancers with potential of reinforcing neuronal networks and thus, formed the basis for the use of humans as test models. Tohda et al. [309] conducted a Japanese version of the Repeatable Battery for Assessment of Neuropsychological Status (RBANS) test on 28 healthy volunteers (between the ages 20 and 81 years) under diosgenin-rich yam extract administration with findings confirming a significant improvement in cognitive function. However, the limitation of this study remains the sample size, non-randomized selection of volunteers (sampled adults were only well-educated Asians) and daily dietary intake and physical activity levels of the individuals were not accessed. 

Studies on animal studies have reported the anti-osteoporosis potential of yam [310,311,312]. Extracts of *D. alata* leaves and roots examined on mouse spleen and bone marrow cells showed the ability of stimulating proliferation on both cells thereby significantly increasing the cell concentrations [312]. Another study on ovariectomized female BALB/C mice revealed that 2 weeks feeding with *D. alata* powder prevented loss of bone mineral density and improved bone calcium status, however, the uterine hypertrophy was not stimulated [313]. Similarly, Han et al. [311] investigated the in vivo effect of ethanol extract of *D. spongiosa* on glucocorticoid-induced osteoporosis in rat. Their findings revealed that *D. spongiosa* extract inhibited glucocorticoid-induced osteoporosis and improved the bone tissue metrology, BMC, BMD and biomechanical indicators. In addition, the authors observed a repair of the microscopic changes of the cancellous and trabecular bones. Based on the changes in the biochemical indexes, these effects were linked to the ability of the yam extract to inhibit excessive bone transition and bone resorption [311]. Other health effects on degenerative diseasessuch as hypertension and osteoarthritis by *Dioscorea* species have been reported [314,315,316].

### 4.8. Yam as an Agent for the Management of Menopausal Symptoms

Menopause is associated with a decline in estrogen level produced by the ovaries resulting in several side effects including mental changes, hot flashes, skin aging, osteoporosis and cardiovascular problems [317]. Hormone replacement therapies (HRT) such as estrogen and progesterone replacement have been deployed to handle these challenges with side effects [318], however, HRT may predispose users to development of degenerative diseases such as ovarian cancer [319]. Rossouw et al. [320] reported an increase in the incidence of coronary heart disease and breast cancer amongst women on estrogen and progestin therapy, hence necessitating alternative treatment options that are as effective and less detrimental. Many traditional systems have implemented treatment plans with a number of plant species for the management of physiological changes associated with menstruation, conception, pregnancy, birth, lactation and menopause [321]. There is reported evidence that *Dioscorea* species, while serving as nutritional supplements, proffer medicinal properties and relief of menopausal symptoms [322]. A Taiwanese study examined the efficacy of *D. alata* in the treatment of menopausal symptoms on 50 women [323]. The authors recorded an evident improvement in the accessed parameters, including feeling tense/nervous or excitable, insomnia, musculoskeletal pain as well as the positive effect of the blood hormone profile among women that received *D. alata.* Similarly, Wu et al. [324] found that replacing two-thirds of staple food with yam for 30 days positively influenced antioxidant status, lipids and sex hormones of 22 apparently healthy postmenopausal women.

Chinese anti-menopausal medicine formula containing rhizomes of *D. oppositifolia* L. have shown the potential to regulate serum levels of estrogen, follicle-stimulating hormone and luteinizing hormone thereby alleviating some side effects in post-menopausal women [325]. This is in line with the study by Lu et al. [244], whose research result supports the use of *D. oppositifolia* in Chinese medicine for easing menopausal disorders. Proteins isolated from *D. alata*, *D. zingiberensis* and *D. oppositifolia* showed potential to upregulate the translational levels of estrogen receptor beta, thus possibly reducing the risk of ovarian cancer [244]. *D collettii* var. hypoglauca have been implicated in the production of herbal formula *feng bei bi xie* used primarily for the treatment of cervical carcinoma which is prevalent within female aging period [326]. In Central America, patients with blood stasis and anemic conditions are treated with a decoction obtained from the rhizomes of *D. bartletti* [207]; while in Latin American communities, the use of decoctions to ameliorate pains of childbirth, painful menstruation, ovarian pains and vaginal cramping have been reported [327]. The diosgenin composition of yam has placed *Dioscorea* species as major constituents for commercial progesterone production used for treatment of menopausal hot flashes [207]. When administered orally to female Sprague Dawley rats, Higdon et al. [328] reported an increase in uterine weight, vaginal opening, vaginal cell proliferation and reduced bone loss. This estrogenic influence mechanism is consistent with the findings of Michel et al. [207] who reported mild in vitro biding affinity for estrogen alpha and beta receptors in their test models. Although anin vitrobioassay does not necessarily correspond toin vivoefficacy, the data seem to implicate a significant influence of *Dioscorea* species in management of issues related to women’s reproductive health.

### 4.9. Yam as Pharmaceutical Excipient

Although much effort have been shown on the importance of yam starch in relation to food, limited attention has been given to its other potentials such as an excipient for the pharmaceutical industry. Zuluaga et al. [329] highlighted that yam starch could be used as a pharmaceutical excipient for tablet and capsule formulation comparable to potato starch, with further potential as thickening agent. Nasipuri [330] reported yam starch as an efficient binder/disintegrant in tablet formulations containing both soluble and insoluble organic medicinal substances. Studies have shown that *D. dumetorum* and *D. oppositifolia* starches are highly compressible and form tablets with acceptable crushing force. Both species possess small granule size, large specific surface, volume-surface mean, surface-number mean and spherical symmetry. These qualities imply better performance as an excipient especially with respect to product process and better homogeneity of mixes when compared with starches from *D. alata* and *D. rotundata*, with larger granules and high amylase content [331,332]. However, under high compression pressures, *D. roundata* and *D. alata* can be used for tablet formulations where faster disintegration and dissolution is desired [332,333].

## 5. Conclusions and Future Perspectives

*Dioscorea* species provide safety nets as foods and conventional and unconventional medicine during famine and endangered periods. Yam constituents such as flavonoid, diosgenin and dioscorin, tannin, saponin and total phenols places them as good food source of bioactive compounds to consumers [23]. However, exploitation of the rich diversity within the *Dioscorea* genus may lead to extinction if proper steps are not taken in terms of advocacy and conservation. This will directly result in loss of this potential source of active compounds for the pharmaceutical industry as well as constituting a huge genetic loss with respect to crop improvement and breeding. Rational and sustainable use is highly encouraged within the array of wild species. Sensible utilization of this diversity entails understanding species availability, ease of access, possibility of preservation, replanting and establishment of priorities in respect to its optimal pharmaceutical use [334]. Plant derived drugs will receive more acceptance in modern medicineand health systems if they can be efficacious, safe and quality controlled as in the case of synthetic products [335]. The understanding of the pharmacologically active compounds within *Dioscorea* diversity will assist in standardizations and analysis of formulations [334]. The gaps in knowledge of chemical composition, ecological factors and geographical spread of diversity and environmental impacts as relates to chemical biodiversity and plant variability need to be urgently addressed.

Investigation to study the medicinal potential of over 600 wild and domesticated *Dioscorea* species requires a multidisciplinary dimension involving indigenous natives who have a thorough grasp of these plants while adopting a well thought out strategy that puts into context society, health, conservation and sustainable use of species biodiversity. Numerous synthetic contraceptives and steroid related hormonal medications are made of dioscin. Unfortunately, the global need of dioscin is around 8000 tons but present production status puts it at 3000 tons [336]. The increasing pressure from pharmaceutical industries is laying a high demand burden thus making this vital resource a scarce commodity looking into the future. Another perspective to this problem is the ineffectiveness of methods for extracting bioactive compounds. It has been reported that the rate of extraction and separation are generally low, with only the extraction of diosgenin accorded priority [337]. It is imperative that efficient strategies that concentrate diosgenin from its natural sources are optimized [144] putting into consideration other compounds and eliminating waste. It is however important to develop carrier systems like nanoparticles for targeted delivery of yam bioactive extracts and compounds, thus improving efficacy while reducing side effects [144]. The need to standardize analytical protocols toward achieving optimal extraction should not be overlooked. Although extraction methods, such as soxhlet, maceration and hydrodistillation, have been extensively applied in the extraction of bioactive compounds, with newly developed methods shown to be much cleaner, higher yielding and efficient, such as thein situ pressurized biphase acid hydrolysis extraction reported by Yang et al. [338] should be explored. The recent technological advancement in chromatographic techniques such as liquid chromatography-mass spectrometry (LC-MS) and high-resolution mass spectrometry (HR-MS) should be utilized for identifying and quantifying the various compounds in yam species. In addition, sophisticated instrumentation such as HR-MS should be applied to unravel possible beneficial unknown compounds in yam crop.

Further in vivo study is highly encouraged with respect to oxidative stress and antioxidant activities using purified compounds isolated from yam species. For most developing and poor countries, it is imperative to diversify into functional foods, including from *Dioscorea* species. These can be consumed on a regular basis thus serving both nutritional and medicinal purposes. These plants, often in the wild, can be targeted for increased production and conservation. The local populace should be enlightened on the consumption values which directly can lead to reduction in the cost of health care while leading to improved diet. However, due to the huge demand by pharmaceutical industries and agencies, most wild *Dioscorea* species are threatened in their natural habitat. The indigenous knowledge and therapeutic potential of most of the *Dioscorea* species is fast eroding as the situation worsens with increasing urbanization, industrialization and over-exploitation. Efforts toward developing comprehensive information on the therapeutic use, dosage and chemical compounds implicated in the treatment of diseases should be accelerated. Most significant is the ascertaining of the safety level and toxicity profiling of these compounds found in undomesticated yams. This will help ease burden in the rural communities that solely depend on these traditional medicine as health remedies. For instance, due to the high cost of steroid based pharmaceuticals in the management of women’s health disorders, the alternative reliance on herbal remedies is the preferred option to treat hormonally regulated health situations in most impoverished communities. Thus, there is need to provide sufficient empirical scientific basis to support the traditional use of the diversity inherent in the yam crop for hormone therapy related treatment.

Recently, the poisoning cases are occasionally reported in association with the rising popularity of *Dioscorea* consumption prescriptions in clinical use. Chronic and excessive exposure to *D. bulbifera* tuber has caused liver injury in some patients [339]. Also, in vivo and in vitro experimental studies have demonstrated that *D. bulbifera* tuber could induce hepatotoxicity [340,341], increase relative liver weight and can cause death [214]. It is noteworthy to mention that concentrated herbal preparations of samples of *Dioscorea* species abound, with most of them having little or no information about the exact composition and required dosage, thereby increasing health risks to potential consumers [214]. It thus becomes imperative to establish estimated toxicity values for *Dioscorea* species towards efficient utilization in food based clinical management. In view of this, further investigation is required and there is need for relevant government and donor agencies to invest in initiatives that support this research direction. A promising option is the application of CRISPR-Cas (clustered regulatory interspaced short palindromic repeats-CRISPR-associated) mediated genome editing to remove toxic or antinutritive compounds in yams. This precision breeding technique has the potential to alter one or more pathways or traits in a given cultivar, more efficiently than conventional breeding and without disturbing the complement of traits for which it is preferred. The current outlook for non-transgenic genome edited crops is that they may avoid the heavy regulatory burden placed on transgenic “genetically modified” plants [342]. The optimization of protocols for genome editing in yam is well underway [343,344] and the availability of more and better yam genome assemblies is proceeding apace [344,345,346,347]. An understanding of the genetic regulation of desirable nutritional and pharmacological compounds can also be leveraged to increase their amounts, especially with increased variety of possible CRISPR-Cas based manipulations. Genome editing is part of a bright future for scientists working to improve the nutritional quality of yams, while making consumption or clinical use safer.

Scientific investigations into the clinical use of *Dioscorea* species with focus on reducing the risk of ovarian cancer, treatment of menopause complications and female ageing diseases needs to focus on characterizing the bioactive compounds and proteins isolated from diverse species. This includes amino acid sequencing, in vivo pharmacokinetic study as well as modulating mechanisms. This will help in the establishment of multi-target based anti-menopausal drug screening, towards developing more effective drug candidates for future use [244]. Furthermore, research to support the use of *Dioscorea* as a therapeutic agent against asthma, urinary tract infections and bladder related complications, rheumatism, arthritis, pelvic cramps and so forth, need to be promoted. Most of the studies have been limited to in vitro and animal models. It is very important to have further insights into the effects of yam on degenerative diseases while putting into consideration the feasibility and long term effects on humans. Limited or no data on safety, toxicity and efficacy as use as contraceptives on human health, during pregnancy, lactation and childhood suggest an issue of concern. The paucity of data on the safety of diosgenin and other bioactive compounds suggests that further investigation should focus on development, toxicity, neurotoxicity and allergenicity. While preclinical and mechanistic findings tend to support the use of diosgenin as a novel, multitarget-based chemopreventive and therapeutic agent against different forms of cancer [288], research should also focus on developing and evaluating standards of evidence. On a commercial scale, the introduction of *Dioscorea* extracts into the growing international market of natural herbs is highly encouraged. The Mexican experience [348] of biodiversity loss of wild Mexican yams should form the basis for conscious sustainable natural resource management especially in Africa as mentioned earlier.

## Figures and Tables

**Table 1:** Proximate compositions of some species of *Dioscorea*.

Species	Proximate Composition (Percentage, %)						
	Moisture	Crude Protein	Crude Fat	Crude Fiber	Ash	Starch	Reference
*D. alata*	64.9–87.8	0.6–18.7	0.23–5.28	0.75–11.0	0.69–8.81	15.6–84.3	[35,36,37,39,42,46,47,48,49,50,51,52,53,54,55,56,57,58,59]
*D. abyssinica*	NR	3.13–5.37	0.31–1.22	1.94–4.91	2.31–3.58	NR	[36]
*D. bulbifera*	61.6–92.5	0.89–15.8	0.30–8.13	0.61–18.2	0.05–8.15	12.5–62.7	[36,37,39,42,43,47,51,58,60,61,62,63,64,65,66]
*D. cayenensis*	62.2–89.4	2.62–6.63	0.27–7.86	0.17–3.26	0.63–5.48	80.75	[36,37,42,48,58,60,67]
*D. delicata*	92.7	0.41	NR	4.87	NR	0.54	[37]
*D. deltoidea*	80.2	1.6	0.2	1.5	0.6	NR	[65]
*D. dodecaneura*	68.4	1.50	NR	NR	NR	18.46	[37]
*D. dumetorum*	64.3–90.2	0.19–10.3	0.37–3.65	0.82–5.65	2.17–7.79	17.0–63.34	[37,42,49,50,60,61,68,69,70,71]
*D. esculenta*	50.65–86.67	5.60 –10.50	0.08–2.58	1.23–7.82	0.25–8.50	17.25	[37,39,42,48,51,72]
*D. fordii*	NR	9.8–10.2	NR	0.92–1.14	NR	75.7–77.1	[35]
*D. hamiltonii*	78.73	4.37	10.2	4.15	8.70	NR	[38]
*D. hispida*	15.8–37.8	1.13–6.20	1.99–9.36	NR	0.29–1.24	11.5	[32]
*D. laxiflora*	82.0	0.26	NR	2.34	NR	8.92	[37]
*D. nipponica*	NR	NR	NR	NR	NR	35.4	[47]
*D. olfersiana*	84.6	0.42	NR	9.53	NR	0.54	[37]
*D. oppositifolia*	78.5–92.1	7.00–13.54	4.40–7.42	4.92–8.47	2.60–6.38	NR	[38,39,51]
*D. pentaphylla*	90.1–93.1	6.48–9.18	4.01–6.24	5.14–7.24	3.36–4.64	NR	[38,39,51]
*D. persimilis*	NR	7.70–8.20	NR	0.88–0.92	NR	68.2–72.2	[35]
*D. piperifolia*	55.4–74.8	2.27–4.38	NR	NR	NR	18.2–26.1	[37]
*D. polystachya*	NR	6.30–12.2	NR	0.99–1.50	NR	60.7–72.5	[35,47]
*D. praehensilis*	64.1	3.64–5.38	0.26–7.83	1.41–3.21	2.13–4.90	NR	[36,42]
*D. pyrifolia*	NR	1.34	NR	NR	0.88	NR	[73]
*D. remotiflora*	78.18	1.91	0.47	1.22	0.85	NR	[74]
*D. rotundata*	54.5–75.2	0.09–8.28	0.09–3.39	0.41–4.33	1.03–4.92	22.0–80.8	[36,37,42,48,49,52,58,72,75,76,77,78,79]
*D. sanpaulesis*	69.2	0.77	NR	10.3	NR	2.62	[37]
*D. sinuata*	75.6	2.32	NR	NR	NR	8.00	[37]
*D. spicata*	81.5–89.3	6.38–8.20	3.26–4.78	4.67–6.31	5.18–5.20	NR	[38,51]
*D. steriscus*	72.5	0.83	NR	16.8	2.06	9.02	[80]
*D. subhastata*	89.0	0.59	NR	0.95	NR	3.69	[37]
*D. tomentosa*	84.5–93.7	5.25–9.54	2.86–6.84	3.21–4.38	2.48–6.53	NR	[38,39,51]
*D. trifida*	69.4–81.3	0.38–6.79	0.03–0.30	NR	0.2–3.37	7.94–64.0	[37,48,81,82]
*D. triphylla*	76.9	2.3	0.2	0.6	0.6	NR	[65]
*D. versicolor*	80.1	1.7	0.2	1.1	0.5	NR	[65]
*D. villosa*	76.4	2.21	6.01	3.50	3.13	NR	[83]
*D. wallichi*	71.1–76.4	10.5–10.8	1.18–3.34	7.48–9.23	6.36–8.42	NR	[39,51]

*D. polystachya*: Chinese yam formerly known as *D. opposita* and *D. batatas*, NR: not reported.

**Table 2:** Mineral composition of yam (*Dioscorea* species).

Species	No of Varieties	Minerals (mg/100g)									Reference
		K	Na	P	Ca	Mg	Cu	Fe	Mn	Zn	
*D. abyssinica* ^1^	13	NR	NR	5.1–56.5	31.02–118.8	NR	NR	20.3–69.7	NR	0.48–0.77	[36]
*D. alata* ^1^	7	1157–2016	52–82.7	117–194	62.5–78.0	64.0–74.6	6.4–6.9	9.9–10.9	3.1–4.3	3.4–4.3	[54]
*D. alata* ^1^	7	240–400	190–380	100–340	20.04–80.2	24.31–97.2	NR	NR	NR	NR	[53]
*D. alata* ^1^	9	NR	NR	NR	31.64–45.3	32.68–47.8	0.42–0.48	0.83–2.2	NR	0.82–2.6	[35]
*D. alata* ^1^	20	1055-2010	8.30–13.1	NR	26.0–53.5	39.0–59.5	NR	NR	NR	1.01–1.8	[57]
*D. alata* ^3^	1	476.8 ± 0.1	68.9 ± 0.02	163.7 ± 0.10	285.8 ± 0.02	116.3 ± 0.69	NR	2.48 ± 0.02	NR	2.12 ± 0.00	[58]
*D. alata* ^3^	2	622.5–742.5	62.5–95.0	219.0–239	6.50–16.50	40.0–41.5	0.10–0.15	1.50–2.00	2.15–2.20	6.65–6.80	[42]
*D. alata* ^1^	16	1055–2010	8.4–13.1	87.8–190.0	26.0–41.0	39.0–58.0	1.23–1.57	NR	0.48–2.21	1.01–1.41	[55]
*D. alata* ^1^	4	NR	NR	26.59–49.12	11.24–120.0	NR	NR	17.75–51.1	NR	0.38–1.18	[36]
*D. alata* ^1^	1	3932.9 ± 0.16	75.4 ± 0.02	NR	3032.1 ± 0.25	120.7 ± 0.005	1.216 ± 0.001	124.3 ± 0.004	1.33 ± 0.001	5.7 ± 0.001	[56]
*D. alata* ^1^	1	5.25 ± 2.12	0.35 ± 0.0	NR	0.22 ± 0.99	0.65 ± 0.71	NR	0.75 ± 0.73	NR	NR	[50]
*D. alata* ^1^	1	786.3 ± 0.14	44.56 ± 0.3	140.14 ± 0.14	448.36 ± 0.11	656.31 ± 0.07	11.20 ± 0.14	24.30 ± 0.19	6.36 ± 0.21	2.26 ± 0.01	[51]
*D. buibifera* ^1^	12	NR	NR	8.72–55.26	15.74–121.3	NR	NR	20.26–90.9	NR	0.4–8.33	[36]
*D. buibifera* ^1^	1	NR	NR	0.521	1410.0	250	NR	NR	NR	NR	[66]
*D. buibifera* ^1^	1	1554.4 ± 0.36	78.24 ± 0.07	154.42 ± 0.53	338.15 ± 0.09	396.20 ± 1.07	2.14 ± 0.04	19.20 ± 0.20	9.40 ± 0.14	1.48 ± 0.03	[51]
*D. buibifera* ^3^	1	525.8 ± 1.41	87.8 ± 0.10	159.5 ± 0.04	378.5 ± 0.10	128.7 ± 0.04	NR	3.14 ± 0.02	NR	2.79 ± 0.01	[58]
*D. buibifera* ^2^	1	560 ± 49	17.8 ± 9.8	61.61 ± 0.8	29.3 ± 4.8	25.9 ± 9.2	0.21 ± 0.03	2.92 ± 0.3	0.35 ± 0.03	0.53 ± 0.06	[65]
*D. buibifera* ^3^	2	1250–1475	92.5–102.5	223.5–224.5	103–116.5	76.5–83.5	0.20	6.00–6.50	1.30–1.35	6.10–6.35	[42]
*D. cayenensis* ^1^	2	NR	NR	19.15–26.12	6.3–27.6	NR	NR	17.2–27.95	NR	0.74–0.75	[36]
*D. cayenensis* ^2^	1	262.3 ± 0.25	8.53 ± 0.05	19.5 ± 0.10	22.53 ± 0.13	61.53 ± 0.25	NR	0.79 ± 0.02	NR	0.39 ± 0.01	[67]
*D. cayenensis* ^3^	1	523.8 ± 0.04	76.8 ± 0.03	167.8 ± 0.02	345.8 ± 0.01	120.2 ± 0.55	NR	2.50 ± 0.08	NR	2.18 ± 0.02	[58]
*D. cayenensis* ^3^	2	700–825	62.5–70.0	164.5–190.5	74.5–80.0	57.5–38.0	0.20	5.0–5.5	1.2–1.25	5.45–5.85	[42]
*D. deltoidea* ^2^	1	340 ± 51	9.12 ± 1.6	33.1 ± 0.6	46.9 ± 6.2	22.8 ± 7.1	0.10 ± 0.0	1.85 ± 1.0	0.31 ± 0.02	0.22 ± 0.04	[65]
*D. dumetorum* ^1^	1	7.03 ± 0.78	0.41 ± 0.14	NR	0.81 ± 0.21	0.95 ± 0.71	NR	0.07 ± 0.14	NR	NR	[50]
*D. dumetorum* ^3^	2	670–772	72.5–77.5	269–286	27.5–29.5	61.5	0.10	2.0–2.50	2.50–2.65	5.80	[42]
*D. dumetorum* ^3^	2	0.03	0.02	NR	0.19–0.21	0.65–0.72	1.36–1.48	0.13–0.16	0.34–0.38	0.03–0.18	[71]
*D. dumetorum* ^3^		NR	NR	151	57.8	NR	NR	8.89	NR	NR	[68]
*D. esculenta* ^3^	2	765–795	87.5–92.5	273.5–294.5	20.5–27.0	67.5–73.0	0.10	2.0	2.70–2.95	6.20–7.80	[42]
*D. esculenta* ^1^	1	1594.3 ± 1.34	86.40 ± 0.14	138.10 ± 0.14	314.01 ± 0.33	436.06 ± 0.54	3.40 ± 0.01	11.48 ± 0.11	5.46 ± 0.11	1.76 ± 0.04	[51]
*D. fordii* ^1^	3	NR	NR	NR	28.56–30.05	34.58–35.63	0.45–0.51	1.82–2.02	NR	1.79–1.85	[35]
*D. oppositifolia* ^1^	1	1431 ± 1.56	102.2 ± 0.54	78.2 ± 0.08	680.6 ± 0.82	432.5 ± 1.11	2.74 ± 0.03	22.0 ± 0.08	6.34 ± 0.01	3.24 ± 0.08	[38]
*D. oppositifolia* ^1^	2	1534–1624	124–168.2	114.1–124.1	294.2–646.2	540.1–634.1	7.62–14.56	32.16–40.76	7.42–9.04	1.56–6.26	[51]
*D. pentaphylla* ^1^	1	1322 ± 2.40	95.2 ± 0.12	96.1 ± 0.06	632.1 ± 0.22	380.0 ± 0.74	12.60 ± 0.14	103.48 ± 0.94	1.32 ± 0.01	3.10 ± 0.01	[38]
*D. pentaphylla* ^1^	1	1441.0 ± 0.98	96.20 ± 0.63	158.18 ± 0.21	444.24 ± 0.09	532.12 ± 0.56	13.26 ± 0.05	66.32 ± 0.14	3.46 ± 0.21	3.42 ± 0.01	[51]
*D. persimilis* ^1^	3	NR	NR	NR	46.55–47.64	46.70–47.42	0.382–0.423	1.73–1.93	NR	1.32–1.45	[35]
*D. polystachya* ^1^	10	NR	NR	NR	39.73–55.82	33.26–54.47	0.35–0.54	1.43–2.58	NR	0.99–2.27	[35]
*D. praehensilis* ^1^	5	NR	NR	20.9–39.0	13.1–118.2	NR	NR	18.36–76.4	NR	0.4–1.09	[36]
*D. praehensilis* ^3^	1	1000 ± 21.2	80.0 ± 7.07	200.5 ± 0.71	79.5 ± 3.54	43.5 ± 0.71	0.40 ± 0.14	9.0 ± 0.0	0.95 ± 0.07	5.4 ± 0.57	[42]
*D. remotiflora* ^2^	1	4891 ± 25	79 ± 6	720 ± 20	242 ± 14	250 ± 10	3.3 ± 0.2	12.4 ± 0.5	4.1 ± 0.2	7.1 ± 0.3	[74]
*D. rotundata* ^3^	2	475–900	70.0–87.5	158–211.5	91.5–103.3	35.5–53.0	0.20–0.25	5.0–6.75	1.15–1.80	6.30–6.80	[42]
*D. rotundata* ^1^	6	NR	NR	26.96–40.21	22.77–114.4	NR	NR	17.75–78.3	NR	0.35–1.02	[36]
*D. rotundata* ^1^	1	1591	10.4	NR	31.0	51.0	NR	NR	NR	1.23	[57]
*D. rotundata* ^3^	3	9.00–71.00	NR	22.00–35.00	2.00–4.00	11.00	NR	1.00	NR	1.00	[76]
*D. rotundata* ^3^	1	530.7 ± 0.10	80.75 ± 0.14	168.7 ± 0.01	278.8 ± 0.15	125.7 ± 0.08	NR	2.88 ± 0.02	NR	2.34 ± 0.00	[58]
*D. rotundata* ^3^	1	209.13 ± 0.03	185.2 ± 0.05	54.00 ± 0.04	132.02 ± 0.04	45.90 ± 0.02	10.06 ± 0.05	81.85 ± 0.01	NR	5.46 ± 0.02	[78]
*D. spicata* ^1^	1	1255 ± 0.48	52.2 ± 0.11	86.1 ± 0.11	172.0 ± 0.21	112.4 ± 0.32	0.78 ± 0.21	22.36 ± 0.38	0.98 ± 0.14	4.18 ± 0.13	[38]
*D. spicata* ^1^	1	1136 ± 0.74	66.34 ± 0.54	166.30 ± 0.27	234.10 ± 0.58	324.16 ± 0.24	7.41 ± 0.11	24.10 ± 0.26	6.70 ± 0.14	2.56 ± 0.04	[51]
*D. tomentosa* ^1^	1	1354 ± 1.34	32.2 ± 0.18	96.1 ± 0.04	272.1 ± 1.01	120.4 ± 0.08	1.34 ± 0.01	24.56 ± 0.04	1.32 ± 0.04	5.20 ± 0.03	[38]
*D. tomentosa* ^1^	1	1245.6 ± 1.14	46.14 ± 0.30	104.06 ± 0.09	266.36 ± 0.16	321.04 ± 0.14	2.46 ± 0.14	28.50 ± 0.07	2.10 ± 0.11	5.40 ± 0.02	[51]
*D. trifida* ^1^	3	830–1350	NR	50.0–120.0	40.0	40.0–50.0	0.67–1.19	NR	NR	0.62–1.79	[81]
*D. triphylla* ^2^	1	317 ± 32	4.15 ± 0.7	56.6 ± 0.1	39.7 ± 8.1	27.3 ± 5.6	0.18 ± 0.05	1.00 ± 0.05	0.25 ± 0.07	0.39 ± 0.1	[65]
*D. versicolor* ^2^	1	250 ± 4	4.91 ± 2.5	40.8 ± 0.2	14.3 ± 1.8	18.3 ± 3.8	0.18 ± 0.02	0.39 ± 0.1	0.14 ± 0.0	0.3 ± 0.06	[65]
*D. villosa* ^3^	1	145.33 ± 1.15	5.40 ± 0.10	43.82 ± 0.49	28.06 ± 4.01	9.47 ± 0.23	NR	NR	0.032 ± 0.0	0.26 ± 0.0	[83]
*D. wallichi* ^1^	1	1361.7 ± 1.01	63.01 ± 0.27	106.40 ± 0.11	748.31 ± 0.32	578.06 ± 0.19	2.46 ± 0.08	20.14 ± 0.04	3.31 ± 0.05	6.66 ± 0.01	[51]

K: potassium, Na: sodium, P: phosphorus, Ca: calcium, Mg: magnesium, Cu: cupper, Fe: iron, Mn: manganese, Zn: zinc, NR: not reported, ^1^ dry weight, ^2^ fresh weight, ^3^ Sample type: not specified, *D. polystachya*: Chinese yam formerly known as *D. opposita* and *D. batatas*.

**Table 3:** Bioactive compound profile of yam (*Dioscorea* species).

Species	Phytochemicals	Reference
*D. alata*	Phenolics, phenol, flavonoid, flavonol, phytates/phytic acid, saponin, oxalates, alkaloid, tannins, allantoin, dioscin, diosgenin, dioscorin, hydrogen cyanide	[35,42,46,48,51,54,58,59,99,123]
*D. bulbifera*	Carotenoid, phenolics, phenol, polyphenol, flavonoid, terpenoid, saponin, steroid, alkaloid, tannins, phytates/phytic acid, oxalates, hydrogen cyanide	[29,42,51,56,58,62,63,64,99,123,124]
*D. belophylla*	Saponins, alkaloids, flavonoids, tannins and phenols	[125]
*D. cayenensis*	Phenolics, phenol, saponin, alkaloid, tannins, phytates/phytic acid, oxalates, dioscin	[29,42,48,58,67,99,126]
*D. deltoida*	Polyphenol	[29]
*D. dumetorum*	Phenols, flavonoid, alkaloid, tannins, phytates/phytic acid, oxalates, dioscorine	[42,70,99,127]
*D. esculenta*	Phenolics, tannins, phytates/phytic acid, oxalates, hydrogen cyanide	[42,48,51]
*D. fordii*	Allantoin, dioscin	[35]
*D. glabra*	Phenol, flavonoid	[123]
*D. hamiltonii*	Phenol, flavonoid	[123]
*D. hirtiflora*	Phenol, flavonoid	[127,128]
*D. hirsute*	Dioscorine	[128]
*D. hispida*	Dioscorine, phenol, flavonoid	[123]
*D. japonica*	Phenols, flavonoilds, glycans	[129,130]
*D. mangenotiana*	dioscin	[126]
*D. oppositifolia*	Phenolics, phenol, flavonoid, tannins, oxalates, hydrogen cyanide	[38,51,123]
*D. panthaica*	Saponins	[131]
*D. persimilis*	Allantoin, dioscin	[35]
*D. pentaphylla*	Phenolics, tannins, oxalates, hydrogen cyanide, phenol, flavonoid	[38,51,123]
*D. polystachya*	Flavones, polyphenols, allantoin, dioscin	[35,132]
*D. praehensalis*	Tannins, phytates/phytic acid, oxalates,	[42]
*D. preussii*	Saponins	[133]
*D. pubera*	Phenol, flavonoid	[123]
*D. rotundata*	Phenolics, phenol, tannins, phytates/phytic acid, oxalates, saponin, alkaloid, hydrocyanatem dioscin	[42,48,58,75,77,79,99,126]
*D. sansibarensis*	Dioscorine	[128]
*D. spicata*	Phenolics, tannins, oxalates, hydrogen cyanide	[38,51]
*D. tomentosa*	Phenolics, tannins, oxalates, hydrogen cyanide	[38,51]
*D. trifida*	Phenolics	[48]
*D. triphylla*	Polyphenol	[29]
*D. versicolor*	Polyphenol	[29]
*D. villosa*	Flavonoid, phenol, saponin, alkaloid, tannins, Phytates/ Phytic acid, oxalates	[84]
*D. wallichi*	Phenolics, tannins, oxalates, hydrogen cyanide, phenol, flavonoid	[51,123]

*D. polystachya*: Chinese yam formerly known as *D. opposita* and *D. batatas*, NR: not reported.

**Table 4:** Medicinal uses of yam (*Dioscorea* species).

Species	Source of Extract	Biological Properties/Administration	Reference
*D. alata*	Tuber/bulb	Cure piles, gonorrhea and leprosy, anti-inflammatory, purgative, diuretic, anti-rheumatic properties; prevent cancer, reduce blood sugar, diabetes	[200,201] ^2^, [202,203] ^1^
	Tuber	Antihelminthic properties	[204,205] ^2^
	Leaf	Fever	[206] ^2^
*D. bartletti*	Rhizome	Stagnation of blood, anemia	[207] ^2^
*D. belophylla*	Tuber	Treatment of fever, dysentery, headache and malaria	[208] ^2^
*D. bulbifera*	Tuber	Treatment of dementia	[209] ^1^
		Treatment of diabetes	[210] ^1^
		Leprosy and tumors	[211,212] ^1^
		Microbial infections and pig cysticercosis	[213] ^1^
		Antispasmodic, analgesic, aphrodisiac, diuretic and rejuvenative tonic	[214] ^1^
		Effects on liver and heart, reduces carbuncles, lung abscesses, breast lumps, goiter	[212] ^1^
		Abdominal pain	[215,216] ^2^
		Cough	[217] ^2^
		Oral contraceptive.	[218,219] ^2^
		Raw tuber consumed as an appetizer	[220] ^2^
		Rheumatism	[221] ^2^
		Aphrodisiac	[222] ^2^
	Aerial bulb	Oxidative stress induced pathological disorders	[210,223] ^1^
		Anthelmintic treatment	[224] ^1^
	Leaf	Treatment of Elephantiasis	[225] ^2^
		Leaf paste fights dermatological diseases	[226] ^2^
	Stem	Fresh stem shoots are used on hair to fight dandruff	[7] ^2^
*D. bellophylla*	Tuber	Lowers blood cholesterol and reduces heart attack	[7] ^2^
*D. cayenensis*	Tuber	Anti-diarrheal	[206] ^2^
*D. collettii*	Rhizome	Cervical carcinoma, urinary bladder carcinoma, renal tumor	[227] ^1^
*D. deltoidea*	Tuber	Digestive disorders, sore throat, diarrhea, abdominal pains, wounds, burns, anemia	[228,229,230] ^2^
		Anti-rheumatic and treatment of ophthalmic conditions	[231] ^2^
		Antihelmintic treatment	[229] ^2^
		Birth control, oral contraceptive	[217] ^2^
		Antihelminthic	[204,232] ^2^
*D. dumetorum*	Tuber	Treatment of diabetes	[233] ^1^
		Control hyperlipidemia, hypercholesterolemia and hyperketonemia	[234] ^2^
		Jaundice treatment	[235] ^2^
*D. esculenta*	Tuber	Inflammations, nervous disorders and respiratory infections	[235] ^2^
		Dysentery and pain relief	[7] ^2^
*D. hamittonii*	Tuber	Stomach ache and appetizer	[235] ^2^
		Management of diarrhea	[236] ^2^
		Piles	[220] ^2^
*D. hirtiflora*	Tuber	Gonorrhea treatment	[127] ^1^
*D. hispida*	Leaf/root/tuber	Treatment of mole, insect bites and insomnia	[225] ^2^
	Tuber	Treatment of vomiting, indigestion and serves as a purgative when consumed fresh	[7] ^2^
		Treatment of wounds and injuries	[219] ^2^
		Ophthalmic ointment	[237] ^2^
*D. japonica*	Rhizome	Diarrhea and dysentery due to spleen deficiency, fatigue, wasting and thirsting, seminal emission, vaginal discharge and frequent urination	[238] ^1^
		Inflammation, asthma, rheumatoid arthritis	[239] ^1^
		coughing and wheezing	[239] ^1^
*D. membranacea*	Rhizome	Cancer	[240] ^1^
*D. nipponica*	Rhizome	Dissipation of lumps and goiter, clears heat, relieves toxicity, cools the blood, stops bleeding and coughing, calms sneezing, poisonous snake bites, bleeding due to blood-heat and whooping cough	[241] ^2^
		Anti-rheumatic, analgesic, aids blood circulation, anti-diuretic, aids digestion	[242,243] ^1^
*D. oppositifolia*	Rhizomes/tuber	Relief of menopausal syndromes, rejuvenation of early mothers	[220,244] ^2^
	Leaf/flower/tuber	Antiseptic for ulcer; the roots are chewed to cure toothache and aphtha	[245,246] ^2^
	Tuber	Increasing fertility in men	[222,236,247] ^2^
		Constituent in epileptic and nasal relief formula	[219] ^2^
*D. panthaica*	Rhizome	Gastric diseases, bone injuries, rheumatic arthritis	[122] ^2^
		Cardiovascular diseases	[248] ^1^
*D. pentaphylla*	Leaf/vine	Treatment of paralysis	[225] ^2^
	Tuber/flower/young shoot	Rheumatism	[7,249] ^2^
	Tuber	Pain relief and reduce swelling	[219] ^2^
		Stomach disorders	[7,250] ^2^
*D. polystachya*	Rhizome	Consumptive cough and dysentery, aid for digestion and gastric motility and for restraining nocturnal emissions	[251] ^1^
*D. prazeri*	Tuber	Antihelminthic	[204] ^2^
*D. pubera*	Tuberous rhizome/bulb	Cure colic pain	[246,252] ^2^
	Tuber	Weakness	[253] ^2^
*D. septembola*	Rhizome	Rheumatism, urethra, renal infection	[254] ^1^
*D. spongiosa*	Rhizome	Rheumatism, urethral, renal infections	[255] ^2^
*D. sylvatica*	Tuber	Decoction used to treat cuts, wounds and sores	[256] ^2^
*D. trinervia*	Tuber	Chronic diarrhea, asthma and diabetes	[7] ^2^
*D. vexans*	Tuber	Anti-fertility	[257] ^2^
*D. villosa*	Rhizome	Rheumatism	[135] ^1^, [258] ^2^
		Menstrual complaints, perimenopausal symptoms	[259,260] ^2^
*D. wallichii*	Tuber	Flatulence and stomach pain	[7,204,261] ^2^
		De-appetizer	[222] ^2^
*D. zingiberensis*	Rhizome	Cough, anthrax, rheumatic heart disease, rheum, arthritis, tumefaction, sprain	[262] ^2^

^1^ Medical study; ^2^ Ethnobotanical/review study; *D. polystachya*: Chinese yam formerly known as *D. opposita* and *D. batatas*.
